# Weekly Liraglutide for the Management of Intractable Polydipsia and Interdialytic Weight Gain in a Patient on Hemodialysis: A Case Report

**DOI:** 10.7759/cureus.102197

**Published:** 2026-01-24

**Authors:** Franklin Mora-Bravo, Pamela T Morales, Gabriela Pincay, Samantha Pineda, Brayan Morales

**Affiliations:** 1 Hemodiafiltration Unit, Pafram Hemodiafiltration Unit, Sucúa, ECU; 2 Department of Nutrition, Pafram Hemodiafiltration Unit, Sucúa, ECU; 3 Department of Nursing, Pafram Hemodiafiltration Unit, Sucúa, ECU; 4 School of Nursing, Universidad Católica de Cuenca, Macas, ECU

**Keywords:** glp-1 receptor agonists, hemodialysis, hypervolemia, interdialytic weight gain, liraglutide, psychogenic polydipsia

## Abstract

Volume status management is a critical challenge in hemodialysis. Water transgression usually results from intractable polydipsia, for which there are no approved pharmacological treatments. Given the emerging evidence on the role of glucagon-like peptide-1 receptor agonists (GLP-1RAs) in modulating neurological pathways that regulate dipsogenic behavior and satiety, their use is proposed as an unprecedented therapeutic opportunity. A 66-year-old woman with end-stage renal disease (ESRD) secondary to glomerulonephritis was in a chronic hemodiafiltration program without residual diuresis. She started hemodialysis in 2017 and, in 2025, developed refractory hypervolemia driven by intractable polydipsia and interdialytic weight gain (IDWG) of 8% (6 L) on one occasion, and, on average, 5.72%. Management was severely limited by persistent baseline hypotension (90/60 mmHg) and episodes of intradialysis hemodynamic instability that prevented the achievement of dry weight. In this scenario, treatment with liraglutide (1.2 mg weekly) was initiated, resulting in a reduction in IDWG to 2.72% and a significant decrease in thirst (from 10/10 to 3/10 on an analog scale), which stabilized blood pressure and improved tolerance to the treatment. GLP-1RAs can act as potent dipsogenic modulators by modulating neural circuits of osmoreception in the central nervous system, independent of glycemic control. Although gastrointestinal effects require proactive management, the systemic enzymatic degradation of liraglutide positions it as a safe alternative in end-stage renal failure. Pharmacological modulation of the neuroendocrine axis of thirst represents a promising therapeutic frontier to transform the management of refractory hypervolemia and improve hemodynamic stability in patients with no residual diuresis without diabetes. The benefit of GLP-1RAs in this case, a non-diabetic patient on hemodialysis with refractory thirst, extends beyond traditional metabolic control. The clinical success of an intentionally chosen weekly liraglutide regimen indicates that the therapeutic goal was to influence the neuroendocrine system that manages thirst and satiety. This case emphasizes that non-adherence to fluid therapy during dialysis can be viewed as a treatable physiological imbalance rather than a behavioral issue. Although there are no standardized guidelines for weekly dosing, this proof of concept points to a potential new treatment approach. Using incretins as tools to regulate thirst deserves further research through controlled trials to verify their long-term effects on hemodynamic stability and quality of life in patients with ESRD.

## Introduction

Fluid management remains one of the greatest challenges for patients with end-stage renal disease (ESRD) undergoing maintenance hemodialysis due to the absolute dependence on replacement therapy to maintain euvolemia. However, interdialytic weight gain (IDWG), defined as the accumulation of fluid between treatment sessions, is closely linked to a patient’s dietary habits. In a prospective multicenter cohort of 1,013 incident hemodialysis patients, researchers found that IDWG ≥4% of dry weight was an independent predictor of major adverse cardiac and cerebrovascular events, even after adjusting for other risk factors [1]. So-called “noncompliant” patients, who systematically exceed these limits, are exposed to aggressive ultrafiltration rates that lead to hemodynamic instability and cumulative organ damage [2]. Fluid noncompliance in patients with IDWG >4% is not merely a failure in dietary behavior but rather the result of persistent physiological and sensory stimuli.

Persistent thirst is the primary driver of excessive intake, mediated by xerostomia, frequently exacerbated by polypharmacy, uremia, and osmotic mechanisms [3]. Despite the high prevalence of hypervolemia and its direct impact on survival, the therapeutic options for its control are remarkably limited. Currently, there is no approved pharmacological treatment for thirst suppression in hemodialysis patients. Management is restricted to behavioral interventions, such as fluid and dietary restriction, with an increasing emphasis on carbohydrate load control. As the oxidative metabolism of carbohydrates produces endogenous water and CO_2_, restricting simple carbohydrates has been proposed as a complementary strategy to reduce IDWG. The oxidation of 100 g of carbohydrates generates approximately 55 to 60 mL of water [4]. Nevertheless, these measures depend entirely on patient adherence.

Glucagon-like peptide-1 receptor agonists (GLP-1RAs), known for their efficacy in managing obesity and eating behavior, appear to exert a modulatory effect that transcends carbohydrate satiety [5-7]. GLP-1RAs, traditionally utilized for their glucose-dependent insulinotropic effects, have recently emerged as potent modulators of fluid balance in the dialysis population [8,9]. Beyond their metabolic role, these agents act upon receptors located in the subfornical organ and the hypothalamus, which are key central regions for thirst regulation. By activating these neural pathways, GLP-1RAs can suppress the dipsogenic (thirst-inducing) drive, providing a novel pharmacological approach to limit excessive IDWG [9]. This mechanism is particularly relevant in hemodialysis patients, where chronic thirst often leads to fluid overload, subsequent cardiovascular strain, and difficult ultrafiltration sessions. Emerging evidence suggests that GLP-1 affects neurological pathways that regulate osmoreception and dipsogenic behavior [8,9]. In this context, the reduction in fluid intake observed in patients treated with these drugs presents an unprecedented therapeutic opportunity for the management of chronically noncompliant patients, and in those for whom there is no approved pharmacological treatment or clinical guideline for the treatment of thirst [10]. This article describes the clinical case of a patient in a hemodialysis program with refractory hypervolemia and IDWG >4%, in whom the use of GLP-1RAs resulted in significant optimization of fluid status and an unprecedented reduction in the subjective perception of thirst.

## Case presentation

The patient was a 66-year-old female with a medical history of hypertension (2012) and stage 5D chronic kidney disease (2014) secondary to crescentic glomerulonephritis. In 2018, she restarted a thrice-weekly hemodialysis program following the loss of a renal graft (deceased donor transplant in 2017 with humoral rejection in 2018). In the initial diagnoses, she was a carrier of grade I obesity, with a body mass index of 33.1 kg/m². She remained without residual diuresis and had a functional aneurysmal fistula in the left upper limb (humerocepahalic); she also presented with chronic vertigo. She was transferred to a hemodialysis service provider unit offering hemodiafiltration near her home in 2019 and has remained on the hemodiafiltration modality ever since. Laboratory tests revealed a normal phosphorus level (3.7 mg/dL) associated with intact parathyroid hormone (iPTH) within the therapeutic range (354 pg/mL). Another relevant finding was hyponatremia (Table 1). The remaining parameters were as expected for a patient in a hemodiafiltration program. A therapeutic plan was established for hemodiafiltration for four hours, three times a week, along with fluid restriction and continued support with erythropoietin and vitamin supplements.

From 2022 to 2024, the patient experienced a highly favorable clinical evolution, characterized by the stabilization of hematological parameters and a marked improvement in her quality of life. Anemia control was achieved (Hb 11.3 g/dL), allowing definitive discontinuation of erythropoietin and intravenous iron while maintaining optimal hemoglobin levels (greater than 11 g/dL) with dietary support alone (Table 1). Despite sporadic episodes of intradialytic pelvic pain associated with hypotension (managed with volume replacement), the patient’s dry weight progressively increased from 70 to 74.5 kg, reflecting active nutritional recovery.

**Table 1:** Evolution of the patient’s laboratory data. AST: aspartate aminotransferase; ALT: alanine aminotransferase

Study date	10/12/21	2/24/22	7/23/22	1/7/23	10/22/23	2/2/24	7/6/24	10/17/24	4/26/25	9/7/25
Survival (months)	74	78	83	89	98	102	107	111	117	122
Age (years)	62	63	63	64	64	65	65	65	66	66
Vascular access (#)	2	2	2	2	2	2	2	3	3	4
White blood cells (4–11 × 10³/µL)	8	7	9	6.8	9.5	7	6.4	8.8	7.7	7.3
Hemoglobin (10–11 g/dL)	10.7	10.7	12.7	11.2	8.6	9.2	8.7	9.1	9.6	11.8
Hematocrit (30–35%)	-	-	39.6	35.8	27.6	30.1	29.2	29.3	30.7	37.9
Mean corpuscular volume (80–100 fL)	78.8	82.5	87.6	81.9	68.7	69.4	87.7	75.5	74.3	80.1
Mean corpuscular hemoglobin (27–33 pg)	24.7	26	28.1	25.6	21.4	21.2	26.1	23.5	23.2	24.9
Platelets (150–450 × 10³/µL)	279	378	282	453	421	438	379	397	340	341
Segmented (1.5–8.0 × 10³/µL)	4.42	3.9	5.23	3.93	6.16	4.22	3.6	5.07	4.94	4.2
Lymphocytes (1.0–4.8 × 10³/µL)	2.31	1.81	2.41	1.9	1.97	1.48	1.72	2.51	1.77	1.78
Ferritin (12–300 ng/mL)	16.2	-	-	13.4	8	-	589.6	12.9	39.9	45
Iron (35–145 µg/dL)	38.4	-	-	43.1	21.8	-	65.9	33.5	39.4	39.8
Transferrin (215–380 mg/dL)	286	-	-	334	379	-	247	332	295	297
Transferrin saturation (20–50%)	13.4	-	-	12.9	5.8	-	26.7	10.1	10	13.4
Prothrombin time (11–13.5 seconds)	-	-	-	12.4	13	11.5	13	13	13.5	12
International normalized ratio (1 UI)	-	-	-	1.24	1.24	1	1.24	1.24	1.33	1.08
TTP (25–35 seconds)	-	-	-	19.5	24.5	16.5	24.5	19.4	23.5	23.5
Glucose (70–99 mg/dL)	98.4	100.9		95.9	95.6	101.5	100.6	124.1	90.5	90.5
Urea (18–55 mg/dL)	161.6	77.3	92.4	76.9	60.4	84.3	77.2	133.6	53.6	109.7
Creatinine (0.7–1.3 mg/dL)	7.86	6.49	6.85	6.18	5.869	5.765	4.909	7.394	4.853	6.146
Cholesterol (<200 mg/dL)	206.5	-	-	258.4	-	-	213.7	-	-	226.4
Triglycerides (<200 mg/dL)	190.4	-	-	228.1	-	-	186.9	-	-	176.2
Albumin (3.5–4.5 g/dL)	4	-	-	4.7	4.5	-	4.8	4.8	4.5	3.8
ALT (7–35 U/L)	20.6	-	-	19	20.6	24.4	29.6	13	24.9	23
AST (8–43 U/L)	17.8	10.3	-	8.2	13.6	19.6	26.6	8.4	13.3	13.7
Alkaline phosphatase(40–129 U/L)	192	239.5	-	614	396.9	-	22.1	423.8	263.9	799.9
Sodium (135–145 mEq/L)	125.4	129.5	130.1	130.7	130	131.2	134.3	130.4	132.6	130.6
Potassium (3.5–5.5 mEq/L)	5.69	5.37	5.9	5.4	5.3	4.8	4.8	4.55	4.54	5.32
Calcium (8.5–10.2 mg/dL)	10.9	9.3	11.5	10.66	9.34	9.4	10.1	10.6	9.1	8.59
Phosphorus (3–5 mg/dL)	3.7	2.62	3.06	3.46	2.26	2.6	2.5	2.58	4.64	4.1
Post-hemodialysis urea (17–43 mg/dL)	30.1	-	-	36.1	-	-	-	16.1	-	-
Post-hemodialysis creatinine (0.9–1.5 mg/dL)	2.1	-	-	2.63	-	-	-	1.18	-	-
Post-hemodialysis sodium (135–145 mEq/L)	132.8	-	-	131.6	-	-	-	132.2	-	-
Post-hemodialysis potassium (3.5–5.5 mEq/L)	3.95	-	-	3.43	-	-	-	3.18	-	-
Parathyroid hormone (150–600 pg/mL)	-	354	-	1066	-	-	352	-	869	833

Functionally, the incorporation of intradialytic exercise and daily walks resulted in a significant change. By the third quarter, she had regained the capacity to perform daily physical activities and reintegrated into home life. Toward the end of the year, vascular access reported excellent extracorporeal blood flow (490 mL/minute). Strict monitoring was maintained due to the presence of chronic arterial hypotension, with a constant systolic blood pressure <90 mmHg, which necessitated the withdrawal of all antihypertensive medication.

In 2025, the patient presented with a critical clinical picture characterized by a vicious cycle of refractory hypervolemia and hemodynamic compromise with persistent hypotension. Current management was severely hindered by baseline hypotension (90/60 mmHg) and intractable polydipsia, resulting in IDWGs of up to 6 L (8% of her dry weight).

**Figure 1:**
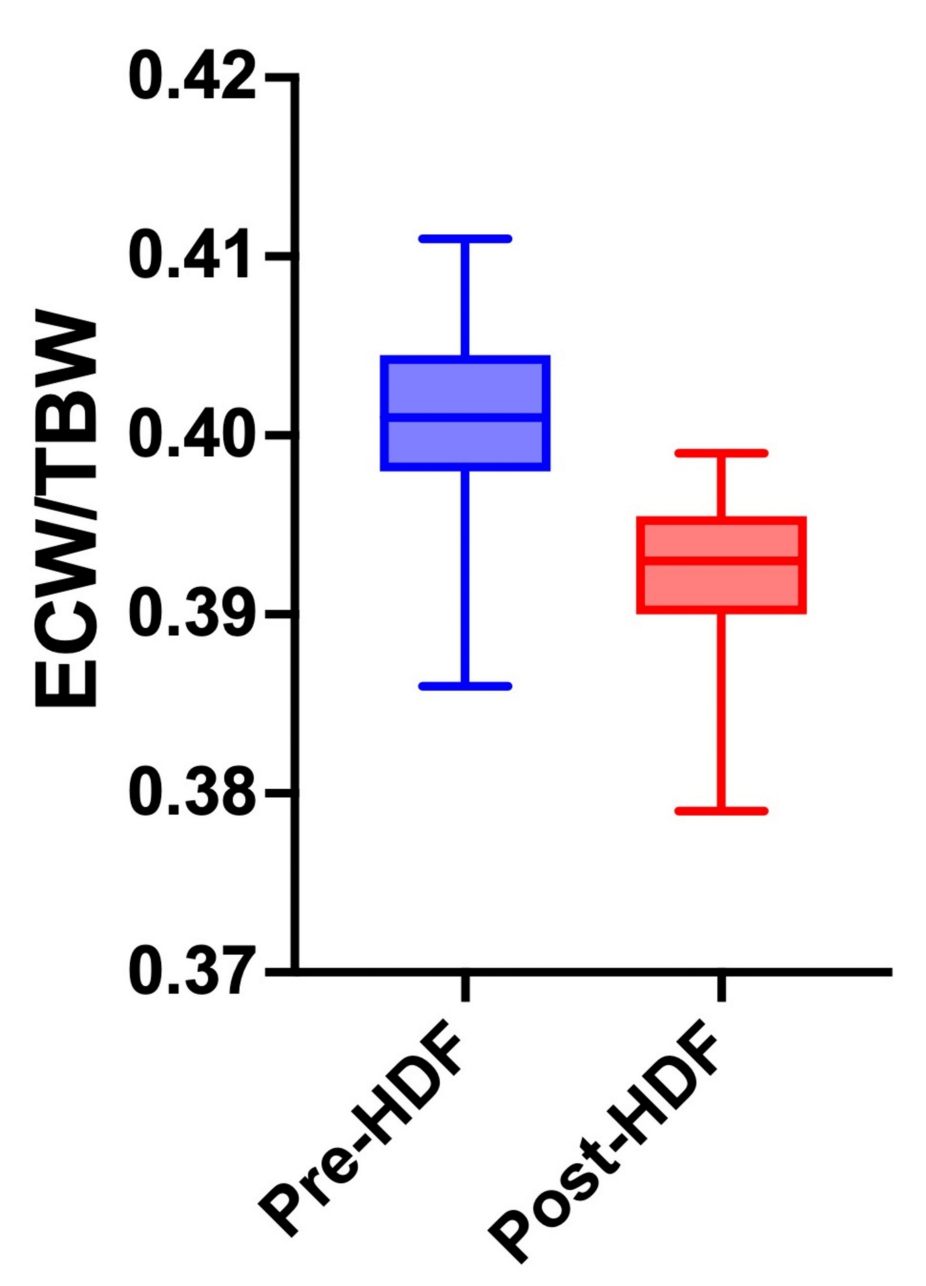
ECW/TBW ratio. The ECW/TBW ratio of less than 0.38 predicts euvolemia. ECW: extracellular water; TBW: total body water; HDF: hemodiafiltration

This fluid overload, confirmed by bioimpedance spectroscopy (extracellular water/total body water ratio of 0.42) (Figure 1), proved impossible to correct during conventional sessions. This was due to episodes of profound intradialytic hypotension (systolic blood pressure of 70 mmHg) without accompanying lipothymia or syncope, which prevented reaching the target dry weight and even necessitated additional rescue sessions on weekends. The patient’s clinical status was further complicated by anemia secondary to small bowel angiodysplasias and a recent postoperative course following rectal fistula repair, both of which aggravated her precarious cardiovascular reserve and limited her tolerance to ultrafiltration.

The cardiac ultrasound ruled out the presence of heart failure but detailed the presence of pericardial effusion (Table 2). In the differential diagnosis considered for the patient’s refractory hypervolemia, we excluded congestive heart failure by clinical assessment and echocardiography (preserved ejection fraction). Hepatic failure was ruled out via normal liver function tests and absence of ascites. Furthermore, the patient’s non-diabetic status and stable blood glucose levels excluded osmotic thirst from hyperglycemia. Finally, despite strict adherence to a low-sodium diet and adjustments in dialysate sodium concentration, the patient’s thirst remained intractable, pointing toward a maladaptive central dipsogenic response rather than a purely dietary or cardiac issue.

**Table 2:** Echocardiography performed on the patient. Heart chambers of normal size and shape. Undilated left ventricle with preserved global systolic function. Slightly decreased right ventricular systolic function. Segmental systolic function at normal rest. It presents slight degenerative changes in the aortic valve. Pericardial effusion is noted. No data on pulmonary hypertension. TAPSE: tricuspid annular plane systolic excursion; LVOT: left ventricular outflow tract

Aortic root (20–37 mm)	33
LVOT diameter (18–24 mm)	25
Left atrium (20–40 mm)	38
Right ventricular diameter (19–34 mm)	33
Diastolic thickness of the septum (6–11 mm)	9
Diastolic diameter of the left ventricular (42–58 mm)	41
Diastolic thickness of the posterior wall (6–11 mm)	11
Posterior wall stroke thickness (<16 mm)	12
Left ventricular end-diastolic volume (46–106 mL)	139
Left ventricular end-systolic volume (14–42 mL)	51
Ejection fraction (50–70%)	63
TAPSE (16–24 mm)	15
Cardiac index (2.5–4 L/minute)	3.9
Pulmonary artery diameter (9–29 mm)	15
Mitral E/A ratio (0.75/1.5)	0.6
Pericardium	Presence of pericardial effusion of 9 mm

Therapeutic intervention

**Figure 2:**
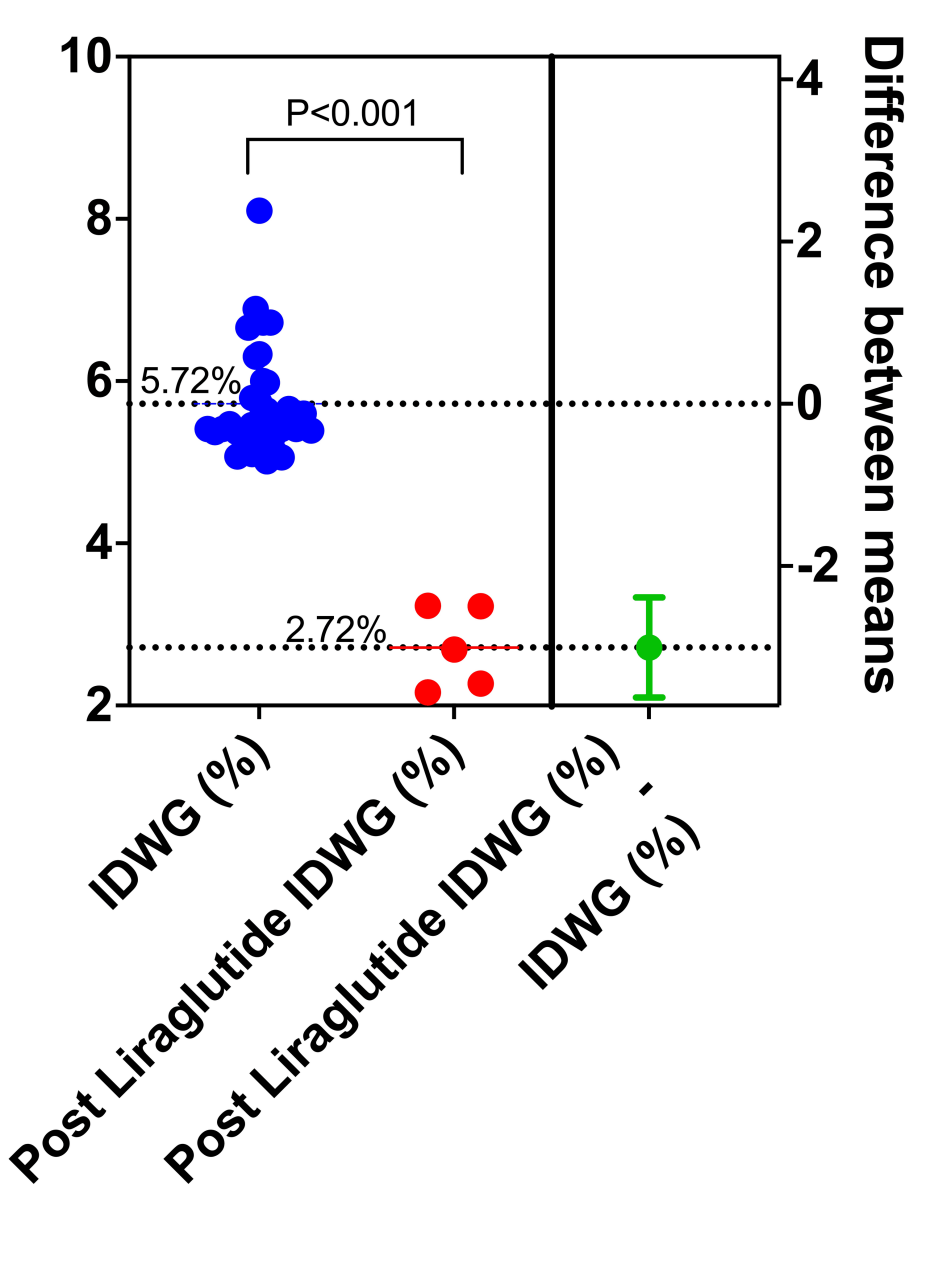
Decrease in IDWG after weekends. The blue dots represent the 33-day IDWG gain data (Mondays) before liraglutide use. The red dots represent the Mondays during liraglutide use. IDWG: intradialytic weight gain

Liraglutide (Saxenda, Novonordisk, 6 mg/mL, 7 USD per dose) was prescribed at a dose of 1.2 mg subcutaneously on the weekends (Fridays) at the end of each hemodiafiltration treatment. Following the first dose, the patient experienced persistent nausea, which was managed with 8 mg of sublingual ondansetron. The patient received one weekly dose every weekend for a total of five weeks. Subjectively, thirst decreased from 10 to 3 on a 1-10 visual analog scale. Carbohydrate consumption also declined, as did the percentage of IDWG, which decreased from 5.72% to 2.72% after the end of the week (Figure 2). The average pre-dialysis weight throughout the period had remained at 78 kg, and the average post-dialysis weight was 74.5 kg. The patient remained on a weekly 1.2 mg regimen of liraglutide. At the eight-week follow-up, the treatment continued to be effective. We observed a minor rebound in fluid intake during the shorter interdialytic interval (Monday to Wednesday), with an IDWG of 4.8%. However, this remained clinically manageable and represented a significant improvement over her baseline, especially during the high-risk weekend interval.

Hemodiafiltration prescription

Hemodiafiltration was performed using Nipro NCU-18 volumetric hemodiafiltration machines. Each session utilized an Elisio 19H filter. The target convection volume was 22 L in post-dilution mode, while the actual convection volume (including replacement and ultrafiltration) reached 26 L. The sodium concentration was set at 135 mEq/L, and the bicarbonate level at 35 mEq/L. The programmed dialysis fluid temperature was 36°C. The dialysate flow rate was maintained at 500 mL/minute. Sodium profiles were not used during any treatment. The dialysate used in each treatment was SFK 203 Glucose (Fresenius Medical Care), which contains potassium at 2.00 mmol/L, calcium at 1.75 mmol/L, magnesium at 0.50 mmol/L, chloride at 109.5 mmol/L, CH3-COO- at 3 mmol/L, sodium at 130 mmol/L, and glucose at 1 g/L. A summary of the monitoring of hemodiafiltration treatments during the period of liraglutide treatment is presented in Table 3.

**Table 3:** Summary of hemodiafiltration treatment during the liraglutide administration period. IDWG: interdialytic weight gain

	Monday sessions, n = 5	Wednesday and Friday sessions, n=10	P-value
Pre-hemodiafiltration weight (kg)	76.3 ± 0.4	77.3 ± 1.0	0.028
Ultrafiltration (L)	2.017 ± 0.377	3.272 ±0.565	<0.001
Post-hemodiafiltration weight (kg)	74.3 ± 0.2	74.2 ± 1.2	0.422
Hemodiafiltration time (minutes)	220 ± 7	218 ± 15	0.370
Kt/v	1.94 ± 0.13	1.91 ± 0.28	0.414
Hemodiafiltration replacement volume (L)	18.99 ± 3.53	19.53 ± 3.41	0.778
Convective volume(replacement + ultrafiltration) (L)	21.00 ± 3.50	22.80 ± 3.54	0.185
Extracorporeal blood flow (QB mL/minute)	471 ± 82	538 ± 65	0.056
Ultrafiltration rate (mL/hour/weight)	7.4 ± 1.5	12.2 ± 1.9	<0.001
IDWG (%)	2.72 ± 1.22	4.40 ± 0.73	<0.001
Initial systolic blood pressure (mmHg)	84 ± 15	85 ± 8	0.435
End systolic blood pressure (mmHg)	86 ± 13	88 ± 11	0.421
Intradialytic hypotension	1 (20%)	3 (30%)	0.680

Patient perspective

By reducing IDWG, dialysis sessions became significantly more tolerable. She no longer experienced drastic blood pressure crashes, which eliminated her fear of treatment. The patient perceived greater ease in adhering to her diet. With the reduction in thirst and carbohydrate cravings, she felt that, for the first time in years, she possessed the tools to meet medical goals without constant suffering. The patient stated, “I feel like my body is no longer desperately begging for water. Dialysis is no longer exhausting because I don’t arrive so swollen, and the fatigue I feel when walking has decreased. Although the medication caused nausea at first, I now feel I have more control over my own illness.”

## Discussion

This clinical case report illustrates the therapeutic potential of GLP-1RAs as a disruptive strategy in the management of refractory hypervolemia in hemodialysis [11-13]. The fundamental finding is liraglutide's ability to modulate subjective thirst perception and carbohydrate intake behavior in patients with hypotension and intractable polydipsia, resulting in a reduction in IDWG from 5.72% to 2.72% over five weeks. Notably, this patient did not have type 2 diabetes mellitus. This approach not only optimized euvolemia but also mitigated the vicious cycle of intradialytic hypotension and ineffective ultrafiltration that characterized the patient’s critical condition. The transition from chronic refractory hypervolemia to sustained hemodynamic stability suggests that modulation of the neuroendocrine axis of dipsogenesis (thirst) and satiety constitutes a promising therapeutic approach, especially relevant in anuric patients with poor adherence to conventional fluid restrictions.

The pathophysiological mechanism underlying the reduction in polydipsia in this case transcended glycemic control, suggesting direct modulation of neural circuits within the central nervous system [9]. The GLP-1 receptor is densely expressed in key hypothalamic regions, including the paraventricular nucleus and the subfornical organ, which act as primary centers for osmoreception and dipsogenic control [11]. The activation of these receptors by liraglutide appears to inhibit angiotensin II-mediated pathways that stimulate thirst, blocking the signaling that induces fluid-seeking behavior [12]. GLP-1RAs modulate the area postrema and the nucleus tractus solitarius, integrating peripheral signals, such as gastric emptying inhibition, with central mechanisms that enhance hedonic satiety. This dual action effectively reduces the cravings for high-carbohydrate foods [13]. In this patient, such neuroendocrine intervention disrupted the positive feedback loop of xerostomia and hyperosmolarity, aligning the perception of fluid satiety with biological requirements and mitigating the behavioral noncompliance that perpetuated her refractory hypervolemia.

Regarding the safety profile, the introduction of GLP-1RAs in patients with ESRD requires close monitoring of gastrointestinal adverse effects. In this case, the onset of persistent nausea following the initial 1.2 mg dose of liraglutide aligns with reports in the literature indicating increased emetic sensitivity in uremic patients due to preexisting delayed gastric emptying and toxin accumulation [11-13]. However, proactive management via sublingual ondansetron has proven to be an effective strategy to facilitate tolerance and ensure therapeutic adherence. Importantly, unlike other renally excreted drugs, liraglutide does not require dose adjustment in end-stage nephropathy because of its systemic enzymatic degradation, positioning it as a safe alternative. Nevertheless, the drastic reduction in IDWG necessitates dynamic adjustments to dry weight and ultrafiltration rates to prevent paradoxical hypotension caused by relative hypovolemia. This requirement underscores that drug safety in this population depends on both gastric tolerance and rigorous post-intervention hemodynamic monitoring.

The transition from clinical observation toward robust research presents a promising horizon in the comprehensive management of renal patients. The findings presented here suggest that the use of GLP-1RAs, especially weekly formulations, could revolutionize thirst control and IDWG in patients with refractory hypervolemia. Future studies should adopt a prospective, multicenter, randomized design, comparing the intervention with placebo in cohorts of chronic noncompliant patients, potentially including oral GLP-1RAs. This approach would not only allow for rigorous quantification of fluid gain reduction and hemodynamic stability via bioimpedance but also validate supervised administration within the dialysis unit as a strategy to ensure adherence. Ultimately, the validation of these therapies could transform clinical practice guidelines, shifting the focus from simple behavioral restrictions toward precise pharmacological modulation of the neuroendocrine axis regulating osmoreception.

We reported in this case an unconventional liraglutide dose that is not recommended for glycemic control in standard populations. However, it served as a successful rescue strategy for fluid management in this specific uremic phenotype. The weekly dosing of 1.2 mg of liraglutide was indeed a deliberate clinical and experimental choice. This decision was based on three pillars: (1) economic accessibility: to ensure long-term treatment feasibility in a resource-limited setting; (2) safety in fragility: to minimize potential gastrointestinal side effects in a patient with advanced uremia; and (3) targeted efficacy: we observed a unique pattern in fluid dynamics. While the patient maintained a low IDWG during the first half of the week, we noted a slight increase in fluid gain toward the second interdialytic period (e.g., Wednesday to Friday). However, this remained clinically manageable compared to her baseline. More importantly, the most critical challenge for dialysis patients is the long interdialytic interval (72 hours) over the weekend, where the highest risk of fluid overload and cardiovascular events occurs. The weekly dose was strategically administered to provide maximum thirst suppression during this high-risk window, effectively transforming a noncompliant behavior into a physiologically controlled state. In ESRD, the conventional daily dosing of liraglutide may not be strictly necessary due to the potential for delayed clearance in uremic states and the patient’s heightened sensitivity to the drug’s central effects on satiety and thirst.

Limitations

In the limitations of this report, we acknowledge that, although we cannot precisely differentiate between mechanisms by which liraglutide causes a decrease in IDWG, they may include the direct dipsogenic modulation (suppression of thirst via the subfornical organ and hypothalamus) or the reduction in metabolic water production due to lower carbohydrate intake (approximately 60 mL of water per 100 g of carbs). As this is a clinical case report rather than a controlled metabolic study, it is difficult to isolate the exact contribution of each mechanism. However, from a therapeutic and pragmatic perspective, both effects are beneficial and were triggered by the initiation of liraglutide. The combined effect was the key to the patient’s clinical improvement. This report does not include a detailed dietary record or a comparison of carbohydrate intake before and after the intervention. Given the nature of this single case report, our analysis was limited to a comparison of mean IDWG between two distinct periods: the baseline period (pre-treatment) and the five-week observation period following the initiation of liraglutide. In a paired t-test to compare the mean IDWG values before and after the intervention, the reduction from 5.72% to 2.72% was found to be statistically significant (p < 0.05), providing further objective weight to the patient’s subjective improvement.

## Conclusions

The benefit of GLP-1RAs in this case, a non-diabetic patient on hemodialysis with refractory thirst, extends beyond traditional metabolic control. The clinical success of an intentionally chosen weekly liraglutide regimen indicates that the therapeutic goal was to influence the neuroendocrine system that manages thirst and satiety. This case emphasizes that non-adherence to fluid therapy during dialysis can be viewed as a treatable physiological imbalance rather than a behavioral issue. Although there are no standardized guidelines for weekly dosing, this proof of concept points to a potential new treatment approach. Using incretins as tools to regulate thirst deserves further research through controlled trials to verify their long-term effects on hemodynamic stability and quality of life in patients with ESRD.
